# 

**Published:** 1849-04

**Authors:** 


### WESTERN JOURNAL

### OF

### MEDICINE AND SURGERY:

EDITED BY

LUNSFORD P. YANDELL, M. D.,

PROFESSOR OF CHEMISTRY AND PHARMACY IN THE UNIVERSITY OF LOUISVILLE.

NO. CXII.—APRIL, 1849.

THIRD SERIES.

Vol. III.....No. IV.

LOUISVILLE, KY:

PUBLISHED MONTHLY BY PRENTICE AND WEISSINGER,

PROPRIETORS AND PRINTERS.

1849.

